# Changes in leukocyte subsets of pregnant gilts experimentally infected with porcine reproductive and respiratory syndrome virus and relationships with viral load and fetal outcome

**DOI:** 10.1186/s13567-014-0128-1

**Published:** 2014-12-14

**Authors:** Andrea Ladinig, Wilhelm Gerner, Armin Saalmüller, Joan K Lunney, Carolyn Ashley, John CS Harding

**Affiliations:** Department of Large Animal Clinical Sciences, Western College of Veterinary Medicine, University of Saskatchewan, Saskatoon, SK Canada; Institute of Immunology, Department of Pathobiology, University of Veterinary Medicine Vienna, Vienna, Austria; Animal Parasitic Diseases Laboratory, Beltsville Agricultural Research Center, Agricultural Research Service, U.S. Department of Agriculture, Beltsville, MD USA; Current address: University Clinic for Swine, Department for Farm Animals and Veterinary Public Health, University of Veterinary Medicine Vienna, Veterinaerplatz 1, 1210 Vienna, Austria

## Abstract

In spite of more than two decades of extensive research, the understanding of porcine reproductive and respiratory syndrome virus (PRRSv) immunity is still incomplete. A PRRSv infection of the late term pregnant female can result in abortions, early farrowings, fetal death, and the birth of weak, congenitally infected piglets. The objectives of the present study were to investigate changes in peripheral blood mononuclear cell populations in third trimester pregnant females infected with type 2 PRRSv (NVSL 97–7895) and to analyze potential relationships with viral load and fetal mortality rate. PRRSv infection caused a massive, acute drop in total leukocyte counts affecting all PBMC populations by two days post infection. Except for B cells, cell counts started to rebound by day six post infection. Our data also show a greater decrease of naïve B cells, T-helper cells and cytolytic T cells than their respective effector or memory counterparts. Absolute numbers of T cells and γδ T cells were negatively associated with PRRSv RNA concentration in gilt serum over time. Additionally, absolute numbers of T helper cells may be predictive of fetal mortality rate. The preceding three leukocyte populations may therefore be predictive of PRRSv-related pathological outcomes in pregnant gilts. Although many questions regarding the immune responses remain unanswered, these findings provide insight and clues that may help reduce the impact of PRRSv in pregnant gilts.

## Introduction

In spite of more than two decades of extensive research, understanding of porcine reproductive and respiratory syndrome virus (PRRSv) immunity is still incomplete. PRRSv is able to persist in infected pigs for several months [1,2] and uses different evasion strategies to circumvent innate and adaptive immune responses, summarized in several reviews [3–5]. Numerous reports have investigated immune responses against PRRSv in vivo including the measurement of cytokine production, the investigation of immune cells, or the measurement of antibody responses. However, a direct comparison of results across different experiments is complicated by several factors including the use of different virus isolates, age and genetics of animals, as well as criteria used to measure immune responses. For the investigation of immune cells by flow cytometry (FCM), additional factors complicating interpretation include methods of data presentation (absolute numbers versus percentages of different cell populations) as well as variation in marker selection used to define leukocyte subsets. In addition, investigations of peripheral blood mononuclear cells (PBMC) subsets in response to PRRSv infection have mainly used nursery or growing pigs in PRRSv respiratory models, whereas reports using pregnant females are sparse. Nielsen et al. [6] inoculated sows at 90 days of gestation to investigate leukocyte populations in piglets surviving in utero infection with PRRSv, but did not characterize leukocyte populations in sows. Christianson et al. [7] investigated peripheral blood leukocytes in sows experimentally infected with PRRSv in mid-gestation. A significant decrease in total leukocytes was found at 3 and 7 days post infection (dpi) and was most pronounced at 7 dpi. Absolute numbers of CD172a^+^ cells, CD1^+^ cells, CD4^+^ and CD8α^+^ T cells were significantly decreased compared to non-infected controls at 3 to 7 dpi; cell counts returned to control levels by 14 dpi [7].

As there are few reports describing changes in PBMC populations in late term pregnant sows or gilts following PRRSv infection, and no studies correlated changes in subpopulations with clinical outcome, the objectives of the present study were to: 1) characterize changes in the major PBMC subpopulations (monocytes, NK cells, B and T cells) of third trimester pregnant gilts following PRRSv infection; 2) analyze phenotypic changes of the major T cell populations (γδ T cells, T helper cells and cytolytic T cells (CTL)) following PRRSv infection; 3) investigate relationships between PBMC subpopulations and viral load in gilt serum and tissues; 4) investigate relationships between PBMC subpopulations and fetal mortality rate defined at the level of the gilt as percent dead fetuses per litter.

## Materials and methods

### Experimental procedures and sample collection

The experimental protocol is described in detail in Ladinig et al. [8]. Briefly, on experimental day 0 (0 days post inoculation; dpi), 114 pregnant Landrace gilts (gestation day 85 (±1)) split over 12 replicates were inoculated (INOC) with PRRSv isolate NVSL 97–7895 (1 × 10^5^ TCID_50_; 2 mL intramuscularly and 1 mL into each nostril), while 19 control gilts were similarly sham inoculated (CTRL). Heparinized blood samples were collected on 0, 2, 6, and 19 dpi, and sera on 0, 2, 6, and 21 dpi. Automated white blood cell (WBC) counts (Z2 Coulter Particle Count and Size Analyzer, Beckman Coulter Inc., FL, USA) and manual differential counts were performed (300 cells total) on heparinized blood samples. On 21 dpi (gestation day 106 ± 1), gilts were humanely euthanized and necropsied. Fetal preservation status was recorded and the percent dead fetuses were calculated for each litter. Samples of lung, tonsil, reproductive (*Lnn. uterini*) and tracheobronchial lymph node from each gilt, as well as a sample of the uterus including adherent fetal placental layers adjacent to the umbilical stump of each fetus were collected and immediately frozen at −80 °C until further processing. The experiment was approved by the University of Saskatchewan’s Animal Research Ethics Board, and adhered to the Canadian Council on Animal Care guidelines for humane animal use (permit #20110102).

### Isolation of PBMC and FCM staining

PBMC were isolated from whole blood samples by gradient centrifugation using lymphocyte separation medium (Ficoll-Paque™ PLUS, GE Healthcare, Mississauga, ON, Canada). Isolated PBMC were counted and transferred into staining buffer (PBS +0.2% gelatin +0.03% sodium azide).

For phenotypic analyses of PBMC, 5 sets of marker panels to detect surface antigens were used in two- or three-color labelling. Table 1 summarizes monoclonal antibodies (mAbs) used to characterize different PBMC subsets. Where commercially available, directly conjugated mAbs were used; otherwise, mAbs specific for CD8β, swine leukocyte antigen-DR (SLA-DR), and the γδ T cell receptor (TCR), produced from hybridoma supernatants at the Institute of Immunology, University of Veterinary Medicine Vienna, Austria, were used in combination with fluorochrome-conjugated, isotype-specific secondary Abs (Table 1). Isotype-matched non-specific antibodies were used as negative controls. PBMC staining was performed in U-bottom 96-well microtiter plates (1 × 10^6^ cells per well). All incubations were performed on ice in the dark. Mastermixes of primary Abs were prepared and 30 μL was added to each well prior to the first 20 min incubation step. After two washes in 200 μL of staining buffer, mastermixes of secondary Abs (10 μL per well) were added and cells were incubated for another 20 min. Finally, cells were washed twice in staining buffer and fixed by resuspension in 200 μL of 2% formaldehyde solution (PBS +2% formaldehyde solution (formaldehyde 37 wt % solution in water stabilized with 7-8% methanol, Alfar Aesar, Ward Hill, MA, USA)).

**Table 1:** **Antibodies used for flow cytometry analyses**

**Antigen**	**Clone**	**Isotype**	**Source**	**Fluorochrome**	**Secondary Ab**
***T-helper cells***					
CD3	BB23-8E6-8C8	IgG2a	BD Biosciences	PerCP-Cy5.5	
CD4	74-12-4	IgG2b	BD Biosciences	FITC	
CD8α	76-2-11	IgG2a	BD Biosciences	Alexa647	
***Cytolytic T cells***					
CD3	BB23-8E6	IgG2b	Southern Biotech	FITC	
CD8β	PPT23	IgG1	In-house		goat anti-mouse IgG1, Alexa647 (Invitrogen)
SLA-DR	MSA3	IgG2a	In-house		goat anti-mouse IgG2a, PE (Southern Biotech)
***γδ T cells***					
Pan-γδ	PPT16	IgG2b	In-house		goat anti-mouse IgG2b, Alexa488 (Invitrogen)
CD2	RPA-2.10	IgG1	AbD Serotec	PE	
CD8α	76-2-11	IgG2a	BD Biosciences	Alexa647	
***NK cells***					
CD3	BB23-8E6-8C8	IgG2a	BD Biosciences	PerCP-Cy5.5	
CD8α	76-2-11	IgG2a	BD Biosciences	Alexa647	
***B cells***					
CD21	B-ly4	IgG1	BD	APC	
CD79α	HM57	IgG1	Dako	PE	
***Monocytes***					
CD172a	74-22-15	IgG1	Southern Biotech	PE	
CD4	74-12-4	IgG2b	BD Biosciences	Alexa647	
CD14	MIL-2	IgG2b	AbD Serotec	FITC	

To stain the intracellular epitope recognized by the CD79α-specific mAb HM57, cells were fixed and permeabilized using a commercial kit (BD Cytofix/Cytoperm, BD Biosciences, Mississauga, ON, Canada) according to the manufacturer’s instructions.

Prior to incubation and after each wash step, cells were resuspended using a plate shaker. For compensation controls, single-stain samples were prepared for each fluorochrome.

### FCM analyses

Stained cells were analyzed using a FACSCalibur flow cytometer (BD Biosciences, Mississauga, ON) equipped with 2 lasers (488 and 635 nm). At least 5 × 10^4^ cells were collected per sample. Results were analyzed using FlowJo, version 7.6.5 (Tree Star, Inc., Ashland, Oregon, USA). Gates were set according to isotype controls and fluorescence minus one (FMO) control samples [9]. The same gate position was used for all samples with a particular marker combination. Cell numbers for each population were corrected by subtracting the number of cells stained by isotype-matched non-specific antibodies. Automated WBC counts and manual differential counts (total number of lymphocytes plus total number of monocytes) were used to calculate the absolute numbers of different PBMC subsets.

### Quantification of PRRSv RNA

PRRSv RNA concentrations were measured in gilt serum collected on 0, 2, 6 and 21 dpi (target log_10_ copies/μL), and in tissues collected at termination (21 dpi) (target log_10_ copies/mg), by strain-specific in-house quantitative reverse transcription polymerase chain reaction (qRT-PCR) as previously described [8].

### Statistical analysis

Separate statistical analyses were conducted using Stata 13 (STATA Corp, College Station, Texas, USA) to address each of the objectives. To meet the key model assumptions, data were log-transformed (log base_10_) as appropriate. Firstly, to determine if major PBMC subpopulations, including major T cell populations, differed between INOC and CTRL gilts over time, multilevel mixed-effects linear regression models were developed. These models used gilt as a random effect and accounted for repeated measures by day. All remaining analyses used data from INOC gilts only. Secondly, potential relationships between PBMC subsets (major PBMC subpopulations and major T cell populations) and PRRS viral load in serum and tissues were analyzed. For these analyses, multilevel mixed-effects linear regression models controlling for experimental replicate were used. Area under the curve (AUC) from 0 to 19/21 dpi was calculated for PRRSv RNA concentration in serum (target copies/μL) and for the total number of each PBMC subset (cells × 10^9^/liter) using the formula AUC = (t_1_-t_0_)(a_1_ + a_0_)/2 + (t_2_-t_1_)(a_1_ + a_2_)/2 + … + (t_n_-t_n-1_)(a_n-1_ + a_n_)/2. The final objective, to determine potential associations between the AUC of PBMC subsets and fetal mortality rate (represented as the percentage of dead fetuses per litter), was analysed using multilevel mixed-effects linear regression models controlling for experimental replicate. To account for multiple comparisons, all associations were considered statistically significant if *P* < 0.01. All final models were evaluated to ensure normality and homoscedasticity of residuals.

## Results

### Changes in total leukocytes and major PBMC subpopulations in response to PRRSv infection

One gilt died (11 dpi) and two gilts aborted (17 dpi, 20 dpi) after PRRSv inoculation; results from those three gilts were excluded from further analysis. Thus, data is presented on 111 INOC and 19 CTRL gilts. With the exception of reduced feed intake and increased rectal temperatures in individual gilts, no severe clinical signs were observed following infection.

**Figure 1:**
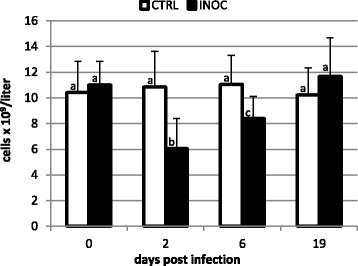
**Changes in total leukocyte counts in response to PRRSv infection in pregnant gilts.** Mean (+SD) total leukocyte counts are presented from 111 INOC and 19 CTRL gilts for the respective study days. Superscript letters indicate significant differences (*P* < 0.001) between INOC and CTRL gilts and between study days within each treatment group.

Changes in total leukocyte numbers in INOC and CTRL gilts following PRRSv infection are displayed in Figure 1. Total leukocyte numbers in INOC gilts decreased 45% from 0 dpi (11.0 ± 1.8 × 10^9^/L) to 2 dpi (6.1 ± 2.3 × 10^9^/L) (*P* < 0.001). Values returned to pre-inoculation levels on 19 dpi. Neutrophil counts (data not displayed in figures) were also significantly decreased in INOC compared to CTRL on 6 dpi (INOC: 2.3 ± 0.9 × 10^9^/L, CTRL: 3.2 ± 1.3 × 10^9^/L, *P* = 0.002) and trended to increase on 19 dpi (INOC: 4.1 ± 2.0 × 10^9^/L, CTRL: 3.0 ± 1.6 × 10^9^/L, *P* = 0.018).

**Figure 2:**
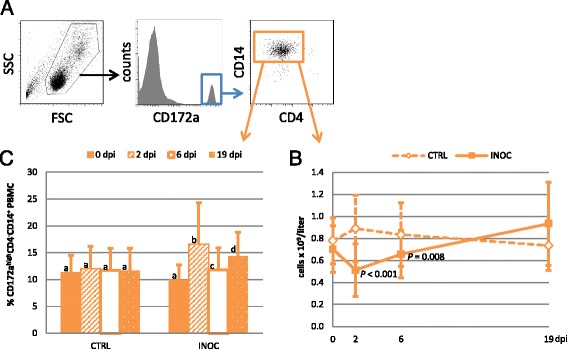
**Changes in monocytes in response to PRRSv infection in pregnant gilts. A)** *Dot plots:* The gating strategy for monocytes (CD172α^high^CD4^−^CD14^+^) is demonstrated using representative data from gilt #53. **B)** *Line chart:* Changes in absolute numbers (mean ± SD) of monocytes (orange) are presented from 111 INOC and 19 CTRL gilts over time. *P*-values indicate significant differences between INOC and CTRL gilts on individual days. **C)** *Bar chart:* The mean percentages (+SD) of CD172a^high^CD4^−^CD14^+^ PBMC from INOC and CTRL gilts are presented for the respective study days. Superscript letters indicate significant differences (*P* < 0.01) between study days within INOC or CTRL gilts.

Monocytes were identified by a CD172a^high^CD4^−^CD14^+^ phenotype (Figure 2A). Total numbers were significantly decreased in INOC gilts compared to CTRL on 2 dpi (*P* < 0.001) and 6 dpi (*P* = 008) (Figure 2B). Since there was a significant increase in the percentage of monocytes within PBMC from INOC gilts on D2 (Figure 2C), the drop in absolute numbers was less severe compared to other PBMC subpopulations (see below). Absolute numbers of monocytes on 19 dpi trended to increase in INOC compared to CTRL gilts (*P* = 0.045) (Figure 2B).

**Figure 3:**
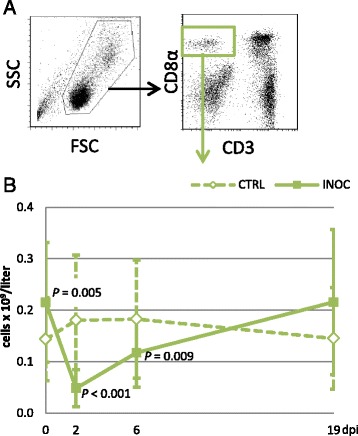
**Changes in NK cells in response to PRRSv infection in pregnant gilts. A)** *Dot plots:* The gating strategy for NK cells (CD3^−^CD8α^+^) is demonstrated using representative data from gilt #53. **B)** *Line chart:* Changes in absolute numbers (mean ± SD) of NK cells are presented from 111 INOC and 19 CTRL gilts over time. *P*-values indicate significant differences between INOC and CTRL gilts on individual days.

NK cells were identified by a CD3^−^CD8α^+^ phenotype (Figure 3A). Their absolute numbers were highly variable in both INOC and CTRL groups. Compared to CTRL gilts, INOC gilts had significantly greater NK cells numbers on 0 dpi (*P* = 0.005), then showed a significant decrease on 2 dpi (*P* < 0.001) and 6 dpi (*P* = 0.009) followed by an increase on 19 dpi that trended towards significance (*P* = 0.015) (Figure 3B). The drop in NK cells from 0 dpi (0.22 ± 0.1 × 10^9^/L) to 2 dpi (0.05 ± 0.04 × 10^9^/L) represented a 73% decrease compared to 0 dpi counts, the most prominent drop of any major PBMC subpopulation.

**Figure 4:**
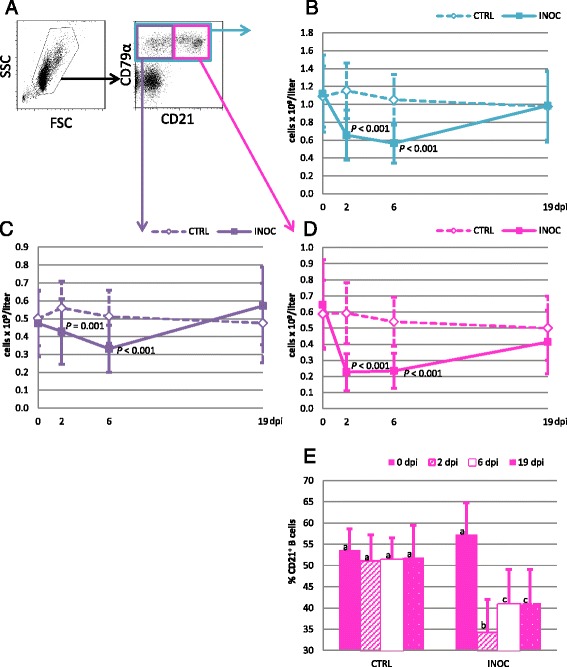
**Changes in B cells in response to PRRSv infection in pregnant gilts. A)** *Dot plots:* The gating strategy for B cells (CD79α^+^) and CD21-defined subpopulations is demonstrated using representative data from gilt #53. **B-D)** *Line charts:* Changes in absolute numbers (mean ± SD) of total B cells (turquois), CD21^−^ (purple), and CD21^+^ (pink) B cells are presented from 111 INOC and 19 CTRL gilts over time. *P*-values indicate significant differences between INOC and CTRL gilts on individual days. **E)** *Bar chart:* The mean percentages (+SD) of B cells expressing CD21 within total CD79α^+^ B cells from INOC and CTRL gilts are presented for the respective study days. Superscript letters indicate significant differences (*P* < 0.01) between study days within INOC or CTRL gilts.

Total B cells were identified by a CD79α^+^ phenotype (Figure 4A, turquois gate). Their absolute counts also decreased significantly on 2 dpi and 6 dpi (*P* < 0.001) in INOC gilts compared to CTRL (Figure 4B). In contrast to the remaining PBMC subpopulations, total B cells did not rebound on 6 dpi, but rather continued to decrease when compared to 2 dpi (0.57 ± 0.2 × 10^9^/L on 2 dpi; 0.66 ± 0.3 × 10^9^/L on 6 dpi), before rebounding to pre-inoculation values on 19 dpi. CD21-defined B cell subpopulations, which can be used to distinguish naïve and activated from effector and memory B cells [10], were also studied (Figure 4A, purple and pink gates). Both B cell subpopulations, CD21^−^CD79α^+^ (Figure 4C) and CD21^+^CD79α^+^ (Figure 4D), were affected. CD21^+^ B cells decreased more rapidly and severely than CD21^−^ B cells, with their absolute numbers dropping to 35% of 0 dpi values by 2 dpi (Figure 4D). By contrast, the CD21^−^ B cell subpopulation in INOC gilts decreased to only 91% and 70% of 0 dpi counts by 2 dpi and 6 dpi respectively, and trended higher than CTRL on 19 dpi (*P* = 0.031) (Figure 4C). In INOC gilts, the percentage of B cells expressing CD21 was significantly lower at all time points after infection compared to 0 dpi (Figure 4E).

**Figure 5:**
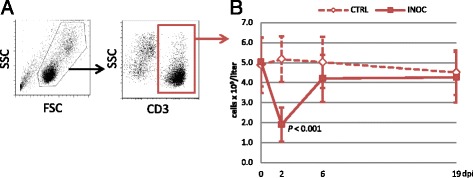
**Changes in T cells in response to PRRSv infection in pregnant gilts. A)** *Dot plots:* The gating strategy for T cells (CD3^+^) is demonstrated using representative data from gilt #53. **B)** *Line chart:* Changes in absolute numbers (mean ± SD) of T cells are presented from 111 INOC and 19 CTRL gilts over time. *P*-values indicate significant differences between INOC and CTRL gilts on individual days.

Total T cells were identified by a CD3^+^ phenotype (Figure 5A). Their absolute numbers decreased significantly in INOC compared to CTRL gilts on 2 dpi (*P* < 0.001), and trended lower on 6 dpi (*P* = 0.013) (Figure 5B). Three subpopulations of T cells, namely γδ T cells, T helper cells, and cytolytic T cells (CTLs), were analyzed in more detail.

### γδ T cells

**Figure 6:**
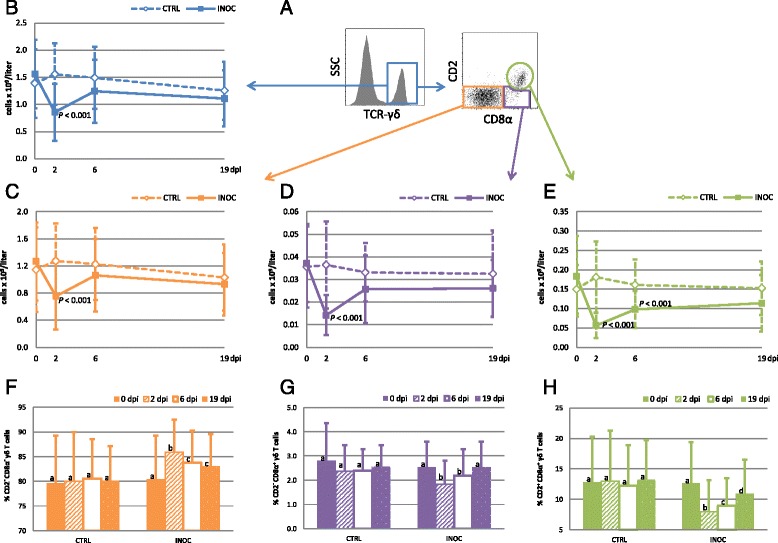
**Changes in γδ T cells in response to PRRSv infection in pregnant gilts. A)** *Dot plots:* The gating strategy for γδ T cells and CD2/CD8α defined subpopulations is demonstrated using representative data from gilt #53. **B-E)** *Line charts:* Changes in absolute numbers (mean ± SD) of total γδ T cells (blue), CD2^−^CD8α^−^ γδ T cells (orange), CD2^−^CD8α^+^ γδ T cells (purple), and CD2^+^CD8α^+^ γδ T cells (green) are presented from 111 INOC and 19 CTRL gilts over time. *P*-values indicate significant differences between INOC and CTRL gilts on individual days. **F-H)** *Bar charts:* The mean percentages (+SD) of CD2^−^CD8α^−^ γδ T cells (orange), CD2^−^CD8α^+^ γδ T cells (purple), and CD2^+^CD8α^+^ γδ T cells (green) within total γδ T cells from INOC and CTRL gilts are presented for the respective study days. Superscript letters indicate significant differences (*P* < 0.01) between study days within INOC or CTRL gilts.

γδ T cells, identified by a mAb recognizing a CD3 molecule associated with the TCR-γδ (clone PPT16) [11], were analyzed for the expression of CD2 and CD8α. This lead to the identification of three distinct phenotypes: CD2^−^CD8α^−^, CD2^−^CD8α^+^, and CD2^+^CD8α^+^ γδ T cells (Figure 6A). Absolute numbers of γδ T cells dropped 45% from 0 to 2 dpi in INOC gilts; 2 dpi counts were significantly lower than in CTRL gilts (*P* < 0.001) (Figure 6B). All three subpopulations showed a significant decrease on 2 dpi compared to CTRL gilts (*P* < 0.001) (Figure 6C to E). While absolute numbers of CD2^−^CD8α^−^ γδ T (Figure 6C) and CD2^−^CD8α^+^ (Figure 6D) γδ T cells did not differ significantly between INOC and CTRL on 6 and 19 dpi, CD2^+^CD8α^+^ γδ T cells (Figure 6E) were significantly lower in INOC on 6 dpi (*P* < 0.001) and also trended lower on 19 dpi (*P* = 0.028). While the percentage of the CD2^−^CD8α^−^ phenotype increased significantly in INOC gilts after infection (Figure 6F), the percentages of both CD2^−^CD8α^+^ (Figure 6G) and CD2^+^CD8α^+^ (Figure 6H) γδ T cells were significantly decreased in INOC gilts.

### T helper cells

**Figure 7:**
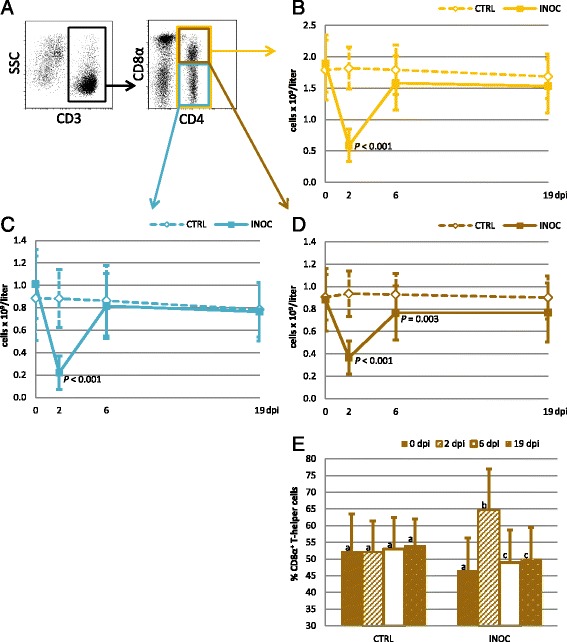
**Changes in T helper cells in response to PRRSv infection in pregnant gilts. A)** *Dot plots:* The gating strategy for T helper cells (CD4^+^) and CD8α-defined subpopulations is demonstrated using representative data from gilt #53. **B-D)** *Line charts:* Changes in absolute numbers (mean ± SD) of total T helper cells (yellow), CD8α^−^ T helper cells (light blue), and CD8α^+^ T helper cells (brown) are presented from 111 INOC and 19 CTRL gilts over time. *P*-values indicate significant differences between INOC and CTRL gilts on individual days. **E)** *Bar chart:* The mean percentages (+SD) of T helper cells expressing CD8α within total CD4^+^ T cells from INOC and CTRL gilts are presented for the respective study days. Superscript letters indicate significant differences (*P* < 0.01) between study days within INOC or CTRL gilts.

Absolute T helper cells, identified by a CD3^+^CD4^+^ phenotype (Figure 7A, yellow gate), were significantly decreased in INOC gilts on 2 dpi (*P* < 0.001) and trended lower on 6 dpi (*P* = 0.044) compared to CTRL (Figure 7B). To investigate their activation/memory status [11], expression of CD8α was analyzed (Figure 7A, brown and light blue gates). Compared to CRTL, INOC gilts had numerically increased numbers of CD8α^−^ T helper cells on 0 dpi (*P* = 0.033), but numbers decreased significantly on 2 dpi (*P* < 0.001) (Figure 7C). The decrease in CD8α^−^ T helper cells in INOC gilts was substantial with counts dropping to 22% (0.22 ± 0.1 × 10^9^/L) of 0 dpi counts. Absolute numbers of CD8α^+^ T helper cells were significantly lower in INOC gilts on 2 dpi (*P* < 0.001) and 6 dpi (*P* = 0.003), and trended lower on 19 dpi (*P* = 0.021) compared to CTRL gilts (Figure 7D). However, given the percentage of T helper cells expressing CD8α increased significantly on 2 dpi (Figure 7E), the drop in total T helper cells on 2 dpi to 42% of the 0 dpi counts was over represented by the CD8α^−^ phenotype, rather than the CD8α^+^ phenotype.

### CTLs

**Figure 8:**
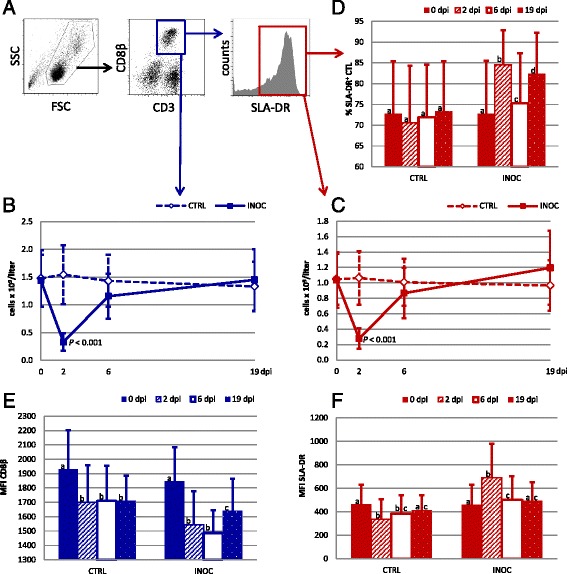
**Changes in CTLs in response to PRRSv infection in pregnant gilts. A)** *Dot plots:* The gating strategy for CTLs (CD3^+^CD8β^+^) and SLA-DR-defined subpopulations is demonstrated using representative data from gilt #53. **B-C)** *Line charts:* Changes in absolute numbers (mean ± SD) of total CTLs (blue) and SLA-DR^+^ CTLs (red) are presented from 111 INOC and 19 CTRL gilts over time. *P*-values indicate significant differences between INOC and CTRL gilts on individual days. **D)** *Bar chart:* The mean percentages (+SD) of CTLs expressing SLA-DR within total CTLs from INOC and CTRL gilts are presented for the respective study days. Superscript letters indicate significant differences (*P* < 0.01) between study days within INOC or CTRL gilts. **E-F)** *Bar charts:* The MFIs of CD8β and SLA-DR on CTLs from INOC and CTRL gilts are presented for the respective study days. Superscript letters indicate significant differences (*P* < 0.01) between study days within INOC or CTRL gilts.

Absolute numbers of CTLs, defined as CD3^+^CD8β^+^ (Figure 8A, blue gate), were significantly decreased in INOC gilts compared to CTRL on 2 dpi (*P* < 0.001) and trended lower on 6 dpi (*P* = 0.013) (Figure 8B). 2 dpi CTL counts in INOC gilts were 77% lower (0.30 ± 0.2 × 10^9^/L) compared to 0 dpi counts. CTLs were further analyzed for the expression of SLA-DR, suggested as a potential marker for activation and antigen encounter [11]. Absolute numbers of SLA-DR^+^ CTLs showed a similar trend and were significantly lower in INOC gilts on 2 dpi (*P* < 0.001) (Figure 8C). Although the percentage of CTLs expressing SLA-DR did not significantly change in CTRL gilts, in INOC gilts the percentage increased significantly to varying degrees on each study day following PRRS infection (Figure 8D). When analyzing the mean fluorescence intensity (MFI) of the three surface markers identified on CTLs, no changes were observed for CD3 (data not shown). The MFI of CD8β decreased significantly from 0 to 2 dpi in both INOC and CTRL gilts (*P* < 0.001), but the drop was more distinct in INOC (Figure 8E). On the other hand, the MFI of SLA-DR significantly increased in INOC gilts on 2 and 6 dpi (Figure 8F), but decreased in CTRL.

### Associations between PBMC subpopulations and PRRS outcome

Detailed results on viral loads (gilt serum and tissues) and on fetal preservation can be found in Ladinig et al. [8]. Briefly, all INOC gilts were viremic on 2 and 6 dpi, and 94/111 (84.7%) remained viremic until termination. The percentages of tissues that tested positive by PRRSv qRT-PCR at termination (21 dpi) in INOC gilts were 90, 99, 99, and 100 for lung, tonsil, tracheobronchial lymph node and reproductive lymph node, respectively. The mean viral loads of positive samples were 3.5 ± 1.2 log_10_ RNA copies/mg in lung, 5.6 ± 0.8 log_10_ RNA copies/mg in tonsil, 5.8 ± 0.8 log_10_ RNA copies/mg in reproductive lymph node, and 4.8 ± 0.9 log_10_ RNA copies/mg in tracheobronchial lymph node. In INOC gilts, the percentage of dead fetuses ranged from 0% to 94.4% (mean 41.0 ± 22.8%).

The total numbers (AUC) from 0 to 19 for monocytes, NK cells, B cells, total T cells, γδ T cells, T helper cells, and CTLs were used to test possible associations with viral load (AUC) and fetal mortality rate. None of the cell subsets were associated with viral load in any gilt tissue. Viral load (AUC) in gilt serum was negatively associated with total T cells (β = −0.140, *P* = 0.007) and total γδ T cells (β = −0.236, *P* = 0.009) over time. None of the analyzed PBMC populations were significantly associated with the percentage of dead fetuses per litter. Total T helper cells (AUC) however, trended to be negatively associated with fetal mortality rate (β = −0.743, *P* = 0.048).

## Discussion

The results presented are part of an extensive and complex dataset obtained in a large scale, multi-institutional project aimed at finding phenotypic and genotypic predictors of PRRSv resistance in pregnant gilts. To investigate genotypic variation in the severity of reproductive PRRS, a large number of gilts were experimentally inoculated in the third trimester of gestation. The number of CTRL gilts was reduced to the minimum to provide baseline data. The objectives of this present study were to investigate temporal changes in the major PBMC and T cell populations and to determine possible associations with measures of PRRS outcome in a reproductive model.

A massive drop in total leukocyte counts was detected early after infection (2 dpi) in all INOC gilts to varying degrees. Leukocyte counts in INOC gilts started to rebound by 6 dpi. Leukopenia has been demonstrated in several other swine viral infections including African swine fever virus (ASFv) [12], classical swine fever virus (CSFv) [13], pseudorabies virus (PRv) [14], and porcine circovirus type 2 (PCV2) [15]. In both ASF and CSF the leukopenia which mainly involved lymphocytes, occurred during the first week after infection and was associated with necrosis and apoptosis of cells [12,13]. Similarly, a significant drop in lymphocytes between 3 and 7 dpi and probably due to apoptosis, was found in pigs infected with H1N2 swine influenza virus. However, the lymphopenia was not accompanied by a drop in total leukocytes [16]. On the other hand, acute PRv infection induced leukopenia involved the loss of up to 40% of monocytes and up to 50% of lymphocytes; the authors hypothesized that besides the killing of infected cells, trafficking of effector cells from the circulation to sites of local infection also contributed to the observed leukopenia [14].

The specific mechanisms associated with the leukopenia observed in this study are speculative and cannot be answered with the current dataset; still this study provides several insightful findings. PRRSv induced apoptosis and necrosis of PRRSv-infected and non-infected bystander cells has previously been reported [17–21]. Cells affected by PRRSv induced apoptosis were mainly macrophages and mononuclear cells, as well as epithelial cells [17,20]. Recently, it was demonstrated that apoptotic cells in thymuses of PRRSv-infected fetuses were predominantly CD3^+^ T cells [22]. Although apoptosis and necrosis might be one possible explanation for the observed leukopenia, in most studies apoptosis and necrosis were observed after 6 dpi [17,20,23]. Moreover, PRRSv was reported to activate anti-apoptotic pathways in macrophages early in in vitro infection models, while PRRSv-infected macrophages die by apoptosis late in infection [21]. For these reasons and given the acuteness of the response and rebound, the authors believe that apoptosis or necrosis of PBMC is unlikely. Moreover, there are no reports in the literature providing evidence of PRRSv replication within lymphocytes.

Therefore, another possible explanation for the leukopenia detected in the present study is an altered trafficking pattern of leukocyte subsets. Interestingly, the current immunological paradigm is that only effector and effector memory cells traffic into non-lymphoid tissue [24]. However, it also is conceivable that PRRSv infection affects entry and exit of lymphocytes from affected lymph nodes as was shown by early experiments in sheep treated with model antigens [25,26]. Obviously, sites of leukocyte migration in our infection model are currently speculative and need to be investigated in future experiments.

The primary sites of PRRSv replication are lung and lymphoid tissues [27]. PRRSv has a tropism to macrophages expressing the receptors sialoadhesin (Sn) and CD163 [28,29]. In the present study, monocytes significantly decreased in INOC gilts on 2 and 6 dpi. However, the drop in absolute numbers was less severe compared to other PBMC subsets. Similar to our findings, Dwivedi et al. [30] reported the frequency of CD172a^+^ cells in PBMC to be significantly decreased 2 days after infection of 7 week-old piglets. A more recent study determined that changes in the absolute numbers of monocytes in PRRSv infected, 6 week-old piglets were virus strain dependent, particularly in the first 2 weeks post-infection. However, at 21 dpi piglets infected with all strains of PRRSv showed significantly increased numbers of monocytes compared to uninfected controls [31]. In the present study, a similar trend of increased monocytes on 19 dpi was observed in INOC gilts.

A prominent drop in NK cells was measured in inoculated gilts early after PRRSv infection. As members of the innate immune system, NK cells possess germ-line encoded, invariant receptors recognizing molecules on the surface of infected or malignantly transformed cells. This makes them particularly important in the early phase of viral infections [32,33]. In contrast to our findings, absolute numbers of NK cells were significantly increased at 2 dpi in 7 week-old piglets in direct contact with pen mates infected with type 2 PRRSv (strain MN 1-18-2). In the same experiment, NK cell numbers were not significantly increased in inoculated piglets [30]. An increase in absolute NK cell numbers was also detected at 10 and 35 dpi in 6 week-old piglets infected with certain European PRRSv isolates [31]. In contrast to European subtype 1 strains, the subtype 3 strain Lena did not induce an increase in NK cell numbers until 35 dpi, rather showing numerically decreased NK cell numbers until 10 dpi. This agrees with our findings. The differences in NK responses among these experiments might be caused by the different virus isolates used, since the results of Weesendorp et al. [31] clearly demonstrated that absolute numbers of NK cells vary between pigs infected with different virus strains.

We detected no association between viral load in gilt tissues and absolute numbers of major PBMC subpopulations or major T cell populations over time. However, increased absolute T cell and γδ T cell counts over time were associated with decreased viral load in gilt serum. This may be relevant for the control of PRRS by selection strategies for breeding programs.

B cells, T helper cells and CTLs with an effector or memory phenotype (CD21^−^, CD8α^+^, SLA-DR^+^, respectively) were less affected by the drop in absolute counts after PRRSv infection. Studies investigating B cell responses towards PRRSv infection mainly measured Ab responses in serum of infected pigs. Only a few reports investigated B cells by FCM; results are somewhat difficult to compare due to differences in marker selection used to define B cells. Nevertheless, Christianson et al. [7] found absolute numbers of CD1^+^ lymphocytes (which represent about 70% of CD79α^+^ B cells; Gerner, unpublished findings) significantly decreased in PRRSv inoculated, mid-gestation sows 7 dpi, which is in accordance with our findings.

Similar to CD21^−^ B cells, total numbers of CD4^+^CD8α^+^ T helper cells dropped less severely than CD4^+^CD8α^−^ T helper cells in INOC gilts on 2 dpi after PRRSv infection. It is well known that many porcine CD4^+^ T helper cells in peripheral blood express CD8α [34] and that both activated and memory T helper cells belong to the CD4^+^CD8α^+^ population [35,36]. This may indicate stronger recruitment of naïve T helper cells from blood to the periphery. A PRRSv specific proliferation of CD4^+^ T cells would likely not occur as early as 2 dpi, but a bystander proliferation of CD4^+^CD8^+^ memory cells may be a non-specific effect of PRRSv infection, and therefore cannot be excluded. From all investigated PBMC populations T helper cells seem to be most relevant for the fetal outcome after PRRSv infection. Although only a trend, absolute numbers of T helper cells over time were positively associated with fetal mortality rate. Therefore, T helper cell counts could be used as a possible indicator of susceptibility to reproductive PRRSv. This has to be confirmed in future experiments.

The fairly high percentage of CTLs expressing SLA-DR in this study agrees with previous data investigating the phenotypic maturation of porcine NK and T cell subsets from birth to six months of age. CTLs in newborn piglets are SLA-DR negative and the first phenotypic change occurs around 3 weeks of age with a massive appearance of SLA-DR^+^ CTLs [36]. In our study, the percentage of SLA-DR^+^ CTLs significantly increased in INOC gilts on 2 dpi. By assuming that SLA-DR expression in porcine CTL is a result of previous antigen contact, this points towards a higher loss of naïve (i.e. SLA-DR^−^) CTLs from blood circulation. But again a non-specific bystander proliferation of antigen experienced CTLs cannot be excluded. A higher decrease of naïve CTLs further corroborated by the decrease in CD8β expression and increase in SLA-DR expression levels (Figure 8E and F) and as such, CD8β^low^SLA-DR^high^ CTLs display the phenotype of terminally differentiated CTLs [37].

In conclusion, PRRSv infection with NVSL 97–7895 in pregnant gilts caused a massive, acute decrease in total leukocyte counts affecting all major PBMC populations, most severely NK cells and CTLs. For all PBMC subsets except B cells, counts started to rebound by 6 dpi indicating a well-functioning immune cell homeostasis, explaining the very mild to absent clinical signs following PRRSv infection in pregnant gilts. However, immune cells migrate extensively and only a very small proportion of immune cells are present in the blood [38], complicating the interpretation of PBMC analyses. That being said, three leukocyte populations may predict relevant biological outcomes in PRRS-infected pregnant gilts. Absolute numbers of total T cells and γδ T cells were negatively associated with PRRSv viral load (AUC of RNA concentration in serum over 21 dpi). Additionally, absolute numbers of T helper cells may predict fetal mortality rate. Although many questions regarding the immune responses remain unanswered, these findings provide insight and clues that may help reduce the impact of PRRSv in pregnant gilts.

## Abbreviations

ASFv: African swine fever virus
AUC: Area under the curve
BVDv: Bovine viral diarrhea virus
CSFv: classical swine fever virus
CD: Cluster of differentiation
CTLs: Cytolytic T lymphocytes
dpi: Days post infection
FCM: Flow cytometry
FMO: Fluorescence minus one
INOC: Gilts inoculated with PRRSv
CTRL: gilts that were sham inoculated
ILL: Innate-like lymphocytes
SLA-DR: Major histocompatibility complex class II
MFI: Mean fluorescence intensity
mAbs: Monoclonal antibodies
NK cells: Natural killer cells
NKR: Natural killer receptor
PBMC: Peripheral blood mononuclear cells
PBS: Phosphate buffered saline
PCV2: Porcine circovirus type 2
PRRSv: Porcine reproductive and respiratory syndrome virus
PRv: Pseudorabies virus
qRT-PCR: Quantitative reverse transcription polymerase chain reaction
Sn: Sialoadhesin
SD: Standard deviation
TCR: T cell receptor
TLR: toll like receptor
WBCs: White blood cells
